# Significance of Neuropilin-1 Expression in Acute Myeloid Leukemia

**DOI:** 10.4274/Tjh.2011.0005

**Published:** 2013-09-05

**Authors:** Tarif H. Sallam, Manal A. Shams Eldin El Telbany, Hanan M. Mahmoud, Mutea A. Iskander

**Affiliations:** 1 Clinical Pathology Department, Faculty of Medicine, Ain Shams University, Cairo, Egypt; 2 Clinical and Chemical Pathology, Faculty of Medicine, Sanaa University, Sanaa, Yemen

**Keywords:** Neuropilin-1, Acute myeloid leukemia

## Abstract

**Objective:** Neuropilin-1 is a vascular endothelial growth factor receptor that acts as a mediator of angiogenesis. Its importance in hematological malignancies such as acute myeloid leukemia (AML) remains to be elucidated. The aim of this study was to evaluate the significance of neuropilin-1 expression in AML patients by both flow cytometry and real-time polymerase chain reaction (PCR) in regard to its diagnostic and prognostic values.

**Materials and Methods:** Bone marrow aspirates of 44 patients with de novo AML and 12 relapsed AML patients were examined in this study. Ten subjects with nonhematological malignancy serving as the control group were also included.

**Results:** Neuropilin-1 expression by flow cytometry showed a highly significant increase in de novo and relapsed AML patients with a mean of 27.1±17.5% and 21.5±16.6%, respectively, compared to control group’s mean of 3.4±1.9%. A cut-off value of 6% was established as differentiating patients from the control group. By real-time PCR, no statistical significance was found in de novo and relapsed AML patients with a mean of 1.9±3.6 IU/L and 0.3±0.2 IU/L, respectively, compared to the control group’s mean of 0.3±0.1 IU/L. Neuropilin-1 surface expression by flow cytometry showed a significant correlation with total leukocyte count and a negative correlation with hemoglobin level in de novo AML patients. In relapsed AML patients, positive significant correlations were found with age, bone marrow blast percentage, and CD14. Neuropilin-1 mRNA level by real-time PCR showed a positive significant correlation with peripheral blood blast percentage and CD117 and a negative correlation with hemoglobin level in de novo AML patients. In relapsed patients, a positive correlation was found with lactate dehydrogenase.

**Conclusion:** Neuropilin-1 can be used as a tool for diagnosis and prognosis in AML patients.

**Conflict of interest:**None declared.

## Abstract (tr)

**Amaç: **Neuropilin-1 anjiyogenez mediatörü olarak rol oynayan vasküler endotelyal büyüme faktörü reseptörüdür. Akut miyeloid lösemi (AML) gibi hematolojik malignitelerdeki önemi tam aydınlatılamamıştır. Bu çalışmanın amacı; AML hastalarındaki hem akım sitometri hem de gerçek-zamanlı polimeraz zincir reaksiyonu (PZR) ile ile tanısal ve prognostik değerleri göz önünde bulundurularak değerlendirilmesidir.

**Gereç ve Yöntemler:** Bu çalışmada 44 de novo AML ve relaps olmuş 12 hastanın kemik iliği aspiratları incelendi. Hematoloji dışı malignitesi olan 10 kişinin örnekleri de kontrol grubu olarak dahil edildi.

**Bulgular:** Akım sitometri ile saptanan neuropilin-1 sunumu, de novo ve relaps AML hastalarında, sırasıyla, ortalama %27.1±17.5 ve %21.5±16.6 bulundu ve kontrol grubuna (ortalama %3.4±1.9) göre son derece anlamlı artış gösterdi. Hastalardan kontrol grubunu ayırmada cut-off değeri %6 olarak belirlenmiştir. Gerçek-zamanlı PZR ile saptanan neuropilin-1 sunumu de novo ve relaps AML hastalarında, sırasıyla, ortalama 1.9 ±3.6 IU/L ve 0.3±0.2 IU/L bulundu ve bu değerler kontrol grubuna (ortalama 0.3±0.1 IU/L) göre istatistiksel olarak anlamlı değildi. De novo AML hastalarında akım sitometri ile saptanan neuropilin-1 yüzey sunumu, toplam lökosit sayısı ile anlamlı korelasyon ve hemoglobin düzeyi ile negatif korelasyon göstermekteydi. Relaps AML hastalarında ise yaş, kemik iliğindeki blast oranı ve CD14 ile aralarında anlamlı korelasyon bulunmuştur. De novo AML hastalarında gerçek- zamanlı PZR ile bakılan neuropilin-1 mRNA düzeyi, periferik kandaki blast yüzdesi ve CD117 ile pozitif anlamlı korelasyon, hemoglobin düzeyi ile de negatif korelasyon göstermiştir. Relaps hastalarda laktat dehidrogenaz ile pozitif korelasyon bulunmuştur.

**Sonuç:** Neuropilin-1, AML hastalarında tanı ve prognozda bir araç olarak kullanılabilir.

## INTRODUCTION

Neuropilins are single-pass transmembrane glycoproteins that were found to be receptors for the vascular endothelial growth factor (VEGF) family of angiogenesis factors, suggesting that the receptor could be involved in blood vessel formation [1,2]. 

 VEGF is an important cytokine that contributes to disease evolution in various myeloid neoplasms. In particular, VEGF has been described as a mediator of leukemia-associated angiogenesis as well as an autocrine growth regulator in leukemic cells [1]. 

In contrast to physiological angiogenesis, the number of studies suggesting a role for neuropilins in human tumor growth and angiogenesis is increasing rapidly [2]. 

Neuropilin-1 (NRP-1) was found to be a receptor or co-receptor for the specific isoform VEGF165 and expressed on endothelial cells (ECs) and several types of tumor cells. VEGF, as a key factor for angiogenesis and tumor growth, exerts its functions mainly through activation of 2 tyrosine kinase receptors: VEGFR-1 (Flt 1) and VEGFR-2 (KDR). Unlike Flt 1 and KDR, NRP-1 does not possess intrinsic tyrosine kinase activity and thus forms a complex with KDR to transmit signals of VEGF. When co-expressed with KDR on ECs, NRP-1 promotes the binding of VEGF165 to KDR and enhances VEGF-mediated mitogenic and chemotactic activity [3]. 

Acute myeloid leukemia (AML) is the most common acute leukemia affecting adults, and its incidence increases with age [4]. Much evidence indicates that bone marrow (BM) neoangiogenesis, orchestrated by different angiogenic growth factors, is implicated in the pathogenesis of AML. VEGF and its receptors are major regulators in neoangiogenesis in AML [5].

Experimental and clinical evidence indicates that BM cells participate in the process of new blood vessel formation. However, the molecular mechanisms underlying their recruitment and their exact role are still elusive. BM cells are recruited to the sites of neoangiogenesis through the NRP-1 receptor and they are essential for the maturation of the activated endothelium and the formation of arteries. The 165-aa isoform of VEGF, which activates the endothelium and recruits NRP-1 myeloid cells, is a powerful arteriogenic agent. In contrast, neither the shortest VEGF121 isoform, which does not bind NRP-1 and thus does not recruit BM cells, nor semaphorin 3A, which attracts cells but inhibits endothelial activation, is capable of sustaining arterial formation [6]. 

Thus, this work was planned to study the diagnostic and prognostic significance of NRP-1 expression in AML patients by both flow cytometry and real-time polymerase chain reaction (PCR). 

## MATERIALS AND METHODS

**Subjects**

This study was carried out on 56 diagnosed AML patients attending the Hematology/Oncology Clinic of the Ain Shams University Hospitals. Forty-four newly diagnosed AML patients (18 males and 26 females) ranged in age from 18 to 70 years with a mean of 39.5±13.1 years. These patients comprised 6 with M0, 10 with M1, 10 with M2, 8 with M3, 2 with M4, 2 with M5, 2 with M6, and 4 with M7 French-American-British (FAB) subtypes. Twelve relapsed AML patients (6 males and 6 females) were also included in this study; their ages ranged from 24 to 70 years with a mean of 44.6±17.2 years. These patients included 2 with M1, 4 with M2, 2 with M4, 2 with M5, and 2 with M6 FAB subtypes; none of them had M0 or M3 FAB subtypes. The diagnosis of AML was based on the standard morphological and immunophenotyping (IPT) criteria [5,6]. Ten subjects with nonhematological malignancy matching the patients in age and sex were recruited to serve as the control group. The study design was approved by the Research Ethics Committee of the Ain Shams University Faculty of Medicine and was conducted according to the Helsinki Declaration of 1975. All samples were taken after obtaining written consent from the patients and the controls.

All subjects and controls underwent the following procedures:

A- Detailed taking of history and clinical examination: Stress was placed on lymphadenopathy and hepatosplenomegaly.

B- Laboratory investigations:

1- Complete blood count (CBC) using a Coulter Counter T660 (Coulter, Hialeah, FL, USA) with examination of Leishman-stained peripheral blood smears for differential leukocyte count and peripheral blood blast count (PB blast %).

2- BM aspiration with examination of Leishman-stained films for bone marrow blasts.

3- IPT analysis and NRP-1 expression of the BM aspirate using a Coulter EPICS XL flow cytometer. The acute leukemia panel of monoclonal antibodies used for IPT was either fluorescein isothiocyanate (FITC)- or phycoerythrin (PE)-labeled. The monoclonal antibodies used were T-cell lymphoid markers (CD2, CD3, CD5, and CD7), B-cell lymphoid markers (CD10, CD19, and CD20), myeloid markers (CD13, CD33, CD14, CD61, CD117, and myeloperoxidase), and common progenitor markers (CD34 and HLA-DR).

4- Detection of NRP-1 by real-time PCR on BM using the SLAN real-time PCR system. 

**Sampling**

Two milliliters of PB was delivered into an EDTA vacutainer tube (1-2 mg/mL blood) for CBC. Four milliliters of BM aspirate was collected from each patient and control under completely aseptic conditions using sterile vacutainers and delivered into 2 EDTA vacutainer tubes. vSamples were divided as follows: 2 mL for IPT and NRP-1 surface expression by flow cytometry and the other 2 mL for RNA extraction, amplification, and NRP-1 gene detection by real-time PCR. 

**Immunophenotyping**

For all of the monoclonal antibodies, including an antibody for the isotype-matched negative control monoclonal antibody, 50 µL of BM sample was pipetted into several tubes. Next, 5 µL of FITC- or PE-labeled MoAbs (provided by Coulter, except for the PE-labeled anti-NRP-1 from R&D Company, UK) was pipetted into the test tube and 5 µL of isotype-matched conjugated immunoglobulins was pipetted into the control tube to determine the nonspecific binding. Cells and monoclonal antibody mixtures were gently vortexed and both tubes were incubated for 15 min at room temperature in the dark. One milliliter of lysing solution was added to each tube for 2-5 min and mixed well. At the end, 1 mL of phosphate-buffered saline was added. The blasts were electronically gated using forward scatter against side scatter. At least 20% of cells should express the marker to be considered positive for the IPT panel, except for CD34, which is considered positive if only ≥10% of cells are positive. For NRP-1 expression, both the percentage and intensity of cells were determined.

**Real-Time PCR of NRP-1**

Total RNA was extracted from the blood specimens using the NucleoSpin RNA blood kit (Macherey-Nagel, Germany) following the manufacturer’s instructions. The total RNA was transcripted to cDNA and amplified using RNA-direct SYBR Green Real Time PCR master mix (Toyobo, Japan). This was done by using a 2X Master Mix for 1-step real-time PCR using a thermostable DNA polymerase derived from Thermus thermophilus (Tth). Tth DNA polymerase exhibits reverse transcriptase activity in the presence of Mn2+ ions. This system allows for 1-step real-time PCR, including reverse transcription and PCR steps. Two micrograms of total RNA was reverse-transcribed into cDNA and the PCR components of the 20 µL of total volume included 10 µL SYBR Green master mix, 2.5 mM Mn, and 0.25 µM of each primer. The sequences of the forward and reverse primers for NRP-1 were AAG ACC TTC TGC CAC TGG GAA CAT and AGT TGC CAT CTC CTG TGT GAT CCT, respectively [7], with a total amplicon of 103 base pairs. The real-time PCR thermal cycler program was done using LG AdvanSure SLAN (LG Life Science, South Korea) at 90 °C for 30 s, reverse transcription at 61 °C for 20 min, and predenaturation at 95 °C for 60 s following 45 PCR cycles of 95 °C for 15 s, 60 °C for 15 s, and 74 °C for 45 s followed by melting curve analysis. The housekeeping β-actin gene was used for normalization. The primer sequences for β-actin were forward CCA AGC CCA ACC GTG AGA AGA T and reverse CAA CGT TCC GTG AGG ATC TTC A. 

**Statistical Analysis **

Data were analyzed using SPSS 16 and are expressed as mean values ± standard deviations. The Kruskal-Wallis test was used for comparison between more than 2 groups and the Wilcoxon rank-sum test was used for comparisons between 2 nonparametric results. The Pearson correlation coefficient test was used for correlations. Receiver operating characteristic (ROC) curves were used to detect the best cut-off levels and Kaplan-Meier curves for survival were calculated.

## RESULTS

**Figure 1:**
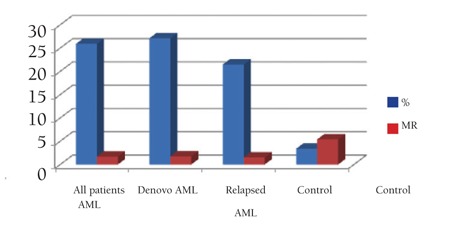
Mean value of NRP-1 (% and MFI) by flow cytometry in studied AML patients

**Figure 2:**
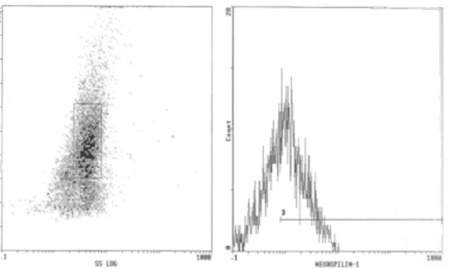
NRP-1 by flow cytometry of acute myeloid leukemia patient. Right: Gated blasts (60%); left: NRP-1 percentage expression (53.6%) and MFI (2.2).

**Figure 3:**
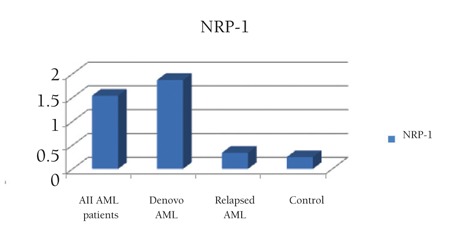
Mean value of NRP-1 by real-time PCR in studied AML patient groups.

**Figure 4:**
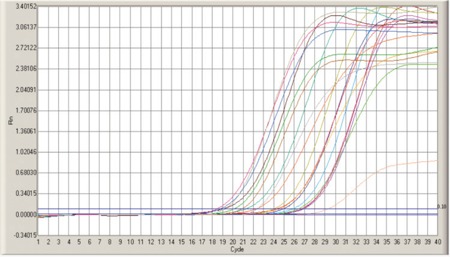
NRP-1 RNA level by real-time PCR in the de novo AML group

**Figure 5:**
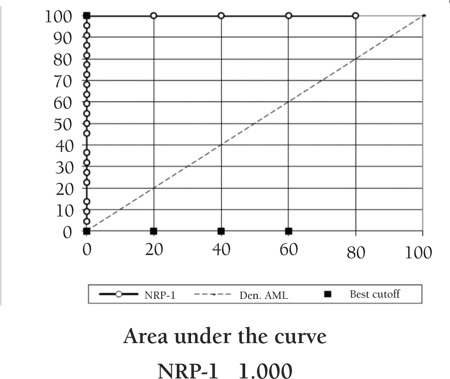
ROC curve analysis showing the diagnostic performance of NRP-1 for discriminating de novo AML from the control by flow cytometry

The results of this study are summarized in Table 1 and Figures 1,2,3,4,5.

Results of NRP-1 by Flow Cytometry (Figures 1 and 2) 

A diagnostic cut-off level of NRP-1 percentage expression was detected in this study, discriminating AML patient groups (either comparing patients as a whole or after dividing patients into de novo and relapsed groups) from the healthy control group, and this level was found to be 6% of cells expressing NRP-1 surface antigen.

NRP-1 percentage expression for all AML cases was 25.9±17.2% taking all patients as a single group, 27.1±17.5% in the group of patients with de novo AML, and 21.5±16.6% in the group of patients with relapsed AML. Mean fluorescence intensity (MFI) levels of NRP-1 was 1.7±0.7 taking all patients as a single group, 1.7±0.8 in the group of patients with de novo AML, and 1.5±0.4 in the group of patients with relapsed AML.

The mean percent positivity of NRP-1 detected by flow cytometry in the control group was 3.4±1.9%, while for MFI the mean level was 5.4±3.9.

There was a highly significant difference in NRP-1 surface expression, both as percent positivity or in its MFI, comparing the control group to patient groups after categorizing patients into de novo and relapsed groups (p<0.001 in all comparisons). However, comparing the de novo and relapsed patient groups, no statistical significance was revealed in NRP-1 expression concerning both percentage and MFI (p=0.401 and p=0.954, respectively) (Table 1).

In de novo AML patients, a highly significant positive correlation was found between NRP-1 percentage expression and total leukocyte count (TLC) (r= 0.577, p=0.005), and a negative correlation was seen between NRP-1 percentage expression and hemoglobin (Hb) levels (r= -0.534, p=0.01), with no significant correlation found between NRP-1 percentage expression and any other parameters including age, platelet count, lactate dehydrogenase (LDH), PB blast %, CD33, CD13, CD14, CD117, CD61, myeloperoxidase, CD2, CD5, CD7, CD34, HLA-DR, CD10, and survival time. 

Relapsed AML patients showed a positive and highly significant correlation between NRP-1 percentage expression and age (r= 0.986, p<0.001) and positive relationships between NRP-1 percentage and both BM blast % and CD14 (r= 0.882,0.899 respectively and p=0.02, 0.015 respectively). However, for other parameters such as TLC, Hb, platelet count, PB blast %, LDH, CD33, CD13, CD117, CD61, myeloperoxidase, CD2, CD5, CD7, CD34, HLA-DR, and CD10, no significant correlations were found. 

Results of NRP-1 by Real-Time PCR (Figures 3 and 4)

The mean value of the NRP-1 RNA level in de novo and relapsed AML patients was 1.9±3.6 IU/L and 0.3±0.2 IU/L, respectively, while the mean level was 0.3±0.1 IU/L in the control group.

No statistically significant difference was found comparing NRP-1 RNA levels between patients and the control group for the 2 groups of de novo patients and relapsed patients (p=0.745 and p=0.661, respectively) (Table 1). 

In the de novo AML patient group, a positive and highly significant correlation was found between NRP-1 RNA level and TLC (r=0.551, p=0.008), as well as between NRP-1 RNA and both PB blast % and CD117 (r= 0.452, 0.451 respectively and p=0.035, 0.035 respectively), but a negative significant correlation was detected with Hb (r=-0.504, p=0.017). There was no significant correlation between NRP-1 RNA level and age, platelet count, LDH, BM blast %, CD33, CD13, CD14, CD61, myeloperoxidase, CD2, CD7, CD34, HLA-DR, CD10, or survival time. 

A positive significant correlation between NRP-1 RNA and LDH (r=0.912, p=0.011) was detected in relapsed AML patients with no other significant correlation between NRP-1 RNA and any other parameters: age, TLC, Hb, platelet count, PB blast %, BM blast %, CD33, CD13, CD14, CD117, myeloperoxidase, CD2, CD5, CD7, CD34, HLA-DR, and CD10.

**Sensitivity and Specificity**

ROC curves (Figure 5) were used to define the best cut-off values and to show the diagnostic performance of NRP-1 in discriminating de novo AML patients from the controls.

**Survival Studies**

Kaplan-Meier curves for overall survival in de novo AML patients were calculated. Survival time for de novo AML patients ranged from 10 to 24 months with a mean of 11.20±0.84. No statistically significant association was found between either survival time or overall survival and NRP-1 expression by either flow cytometry or real-time PCR. 

## DISCUSSION

AML is a heterogeneous group of diseases characterized by uncontrolled proliferation of clonal neoplastic hematopoietic precursor cells and impaired production of normal hematopoiesis [8]. Angiogenesis plays a significant role in the pathogenesis of AML and in the mechanism of disease progression [9]. 

NRP-1 is a VEGF co-receptor and is commonly over-expressed in regions of physiological and pathological angiogenesis, but neuropilin-mediated gene expression and the definitive role of neuropilins in angiogenic processes are not fully characterized [10]. 

The aim of this study was to evaluate the significance of NRP-1 expression in AML patients by both flow cytometry and real-time PCR. Fifty-six AML patients were studied for detection of NRP-1 levels both by flow cytometry (presented as percentage expression and MFI) and its RNA level as estimated by real-time PCR.

Patients included in this work were classified for better evaluation into 2 groups: de novo and relapsed AML cases.

All patients expressed NRP-1 by flow cytometry and the cut-off level for AML diagnosis was determined to be 6% positivity. This study is one of the few that address the issue of NRP-1 in AML. Comparison of patients’ NRP-1 percent positivity or MFI to those of the control group showed a highly significant difference between groups. However, by real-time PCR, no statistical significance was found while comparing patients to controls.

Consistent with our results, a study done by Schuch et al. [7] found that NRP-1 was expressed in acute myeloid leukemic cells. Kreuter et al. [9] reported that the expression of NRP-1 was increased in the bone marrow of AML patients.

Lu et al. [3] examined mRNA expression of NRP-1 in leukemic cells and found that it was increased in AML patients compared with healthy controls. NRP-1 expression was directly correlated with myeloblast percentage in the bone marrow of patients with AML, suggesting that NRP-1 correlates with tumor load.

Staber et al. [11] demonstrated that recurrent AML is commonly associated with mRNA expression changes in a set of 58 genes, such as the angiogenic molecules’ fibroblast growth factor receptor-1 and thrombospondin-2. The discrepancy between our results regarding NRP-1 by real-time PCR and those of other researchers may be because the characterization of NRP-1 by real-time PCR is not standardized, leading to difference in results.

Similar to studies done by Lu et al. [3] and Kreuter et al. [9], no statistical correlation was found between NRP-1 expression and sex, hepatomegaly, splenomegaly, or lymphadenopathy. However, in this study, NRP-1 percentage expression by flow cytometry showed a high positive correlation with age in relapsed patients. 

Studying NRP-1 expression in relation to prognosis, there was a highly positive and significant correlation between it and TLC in de novo AML patients as measured either by flow cytometry or real-time PCR, which Lu et al. [3] failed to detect using PCR techniques. 

A negative correlation was found here between both NRP-1 expression by flow cytometry and NRP-1 RNA by real-time PCR and Hb concentration in de novo AML cases. In contrast, Lu et al. [3] reported no correlation between NRP-1 RNA as measured by real-time PCR and Hb concentration.

A significant positive correlation was detected between NRP-1 percentage expression and BM blasts in relapsed cases. Another positive correlation was found between NRP-1 RNA and PB blasts in de novo cases. This was in agreement with Lu et al. [3], who reported that increased NRP-1 expression was directly correlated with the blast percentage in the PB of AML patients.

In relapsed cases, a significant correlation was found between NRP-1 percentage expression and CD14. This was in agreement with the findings of Bradstok et al. [12], who reported that correlation with CD14 had a poorer outcome in AML patients

There was a positive correlation between NRP-1 expression and LDH. Wimazal et al. [13] reported that a progression to AML was diagnosed in 60% of patients with increasing LDH. They also showed that an increase in LDH over time is associated with a higher probability of AML evolution and a reduced probability of survival, and so they recommended using LDH as a prognostic follow-up parameter in AML.

The current study showed a significant positive correlation between NRP-1 and CD10 percentage expression. However, in a study by Webber et al. [14] excluding t(15;17) cases, they showed association between CD10 percentage expression and complete remission of AML patients.

In a study by Younan et al. [15] on Egyptian AML patients using real-time quantitative RT-PCR, it was revealed that NRP-1 was expressed in 95% of AML cases with levels higher in patients than controls, and there was a statistically significant difference in NRP-1 levels between patients who went into complete remission and those who did not. They concluded that NRP-1 is significantly associated with acute leukemia and that its level might serve as an indicator for disease severity and progression. 

Anti-VEGF antibodies have received much attention lately for their ability to block tumor angiogenesis and prolong the life of cancer patients [16]. In 2004, bevacizumab (Avastin), a humanized monoclonal antibody against VEGF-A, became the first antiangiogenic drug approved by the US Food and Drug Administration as a first-line treatment for metastatic colorectal cancer in combination with chemotherapy. Another anti-VEGF antibody, ranibizumab (Lucentis), a monoclonal antibody Fab, has been successful in the treatment of neovascularization associated with wet neovascular age-related macular degeneration, thereby alleviating blindness in patients [17]. However, in cancer patients, the anti-VEGF–chemotherapy combination has had adverse effects, including hypertension, impaired wound healing, and arterial thrombotic events [18]. Thus, a promising development surfaced, whereby it was reported that antibodies to NRP-1 in combination with anti-VEGF enhanced the ability of anti-VEGF to block tumor growth [19].

Silencing of the NRP-1 gene results in a significant decrease of VEGF- induced cell proliferation and migration in HEL cells, suggesting the role of VEGF in leukemia progression via its newly identified receptor, NRP-1. Therefore, the blocking of NRP-1 signaling may represent a novel therapeutic approach for the treatment of a subset of AML [3]. 

In conclusion, NRP-1 by flow cytometry can be used as a diagnostic tool for AML diagnosis. It can also be used to detect prognosis by both flow cytometry and real-time PCR techniques.

## CONFLICT OF INTEREST STATEMENT

The authors of this paper have no conflicts of interest, including specific financial interests, relationships, and/ or affiliations relevant to the subject matter or materials included.

## Figures and Tables

**Table 1:**
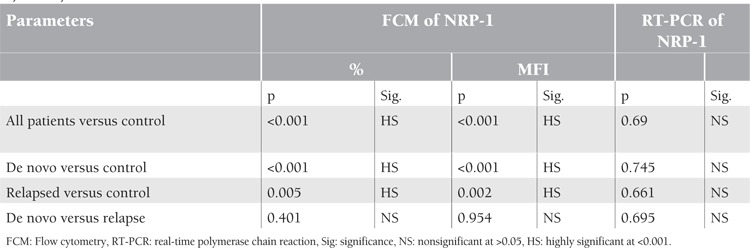
Comparison between different patient groups and the control group as regards NRP-1 expression by flowcytometry and RT-PCR
